# A Novel Protein Elicitor BAR11 From *Saccharothrix yanglingensis* Hhs.015 Improves Plant Resistance to Pathogens and Interacts With Catalases as Targets

**DOI:** 10.3389/fmicb.2018.00700

**Published:** 2018-04-09

**Authors:** Yanan Zhang, Xia Yan, Hongmei Guo, Feiyang Zhao, Lili Huang

**Affiliations:** ^1^College of Life Sciences, Northwest A&F University, Yangling, China; ^2^State Key Laboratory of Crop Stress Biology for Arid Areas, Northwest A&F University, Yangling, China; ^3^College of Plant Protection, Northwest A&F University, Yangling, China

**Keywords:** protein elicitor, induced systemic resistance, catalase, molecular mechanism, biocontrol microbe

## Abstract

Previously, we reported the biocontrol effects of *Saccharothrix yanglingensis* strain Hhs.015 on *Valsa mali*. Here, we report a novel protein elicitor BAR11 from the biocontrol strain Hhs.015 and its functions in plant defense responses. Functional analysis showed that the elicitor BAR11 significantly stimulated plant systemic resistance in *Arabidopsis thaliana* to *Pseudomonas syringae* pv. *tomato* DC3000. In addition, systemic tissues accumulated reactive oxygen species and deposited callose in a short period post-treatment compared with the control. Quantitative RT-PCR results revealed that BAR11 can induce plant resistance through the salicylic acid and jasmonic acid signaling pathways. Further analysis indicated that BAR11 interacts with host catalases in plant cells. Taken together, we conclude that the elicitor BAR11 from the strain Hhs.015 can trigger defense responses in plants.

## Introduction

Pathogen infection has caused huge losses in agricultural production. Plants use different regulatory mechanisms such as physical and chemical barriers to protect themselves from pathogens, insect pests, and adverse abiotic stresses (Fu and Dong, 2013). Biotic or abiotic stress elicits plants’ innate immunity and defense responses. In addition to these non-specific defense mechanisms, there are two other major types of induced resistance: systemic acquired resistance (SAR) induced by pathogenic microorganisms, and induced systemic resistance (ISR) induced by rhizobacteria (Pieterse et al., 2014). SAR and ISR provide broad-spectrum, systemic, and non-specific resistance. Plants have evolved sophisticated mechanisms for defense against necrotrophic and biotrophic pathogens (Zheng et al., 2012). Precise, complex regulation of phytohormones, including salicylic acid (SA) and jasmonic acid (JA), facilitates successful defense against these pathogens. In general, plants activate SA-mediated defense against biotrophic pathogens and JA-induced defense against herbivorous insects or necrotrophic pathogens with a few exceptions (Spoel and Dong, 2008). The SA defense pathway plays an essential role in plant defense against biotrophic pathogens, whereas the JA defense pathway is involved in plant defense against herbivores and necrotrophic pathogens (Pieterse et al., 2014).

Plant growth-promoting rhizobacteria (PGPR) can stimulate resistance responses by releasing elicitors, which are substances that simulate plants to produce a defensive reaction, thereby achieving control over plant diseases (Chisholm et al., 2006). These elicitors belong to a diverse range of molecular types by detecting pathogenic- or microbe-associated molecular patterns (PAMPs or MAMPs), and can be proteins, polypeptides, oligosaccharides, and lipids. Elicitor proteins are usually derived from pathogens such as Gram-negative bacteria (Baillieul et al., 1995) and oomycetes (Amelot et al., 2011), and only a few have been isolated from PGPR. Elicitors can induce a series of plant responses and then activate signaling cascades and changes. Reactive oxygen species (ROS) and nitric oxide (NO) are also formed in some tissues as response. Many of the ISR-inducing microbes identified until date have been Gram-negative bacteria belonging to the genera *Pseudomonas* and *Bacillus* (Jiang et al., 2016). Dimethyl disulfide from *Bacillus cereus*, volatile chemicals from *B. subtilis* and *B. amyloliquefaciens*, and PeBA1 from *B. amyloliquefaciens* are all reported to trigger plant defense responses (Ryu et al., 2004; Huang et al., 2012; Wang et al., 2016). Currently, knowledge of protein elicitors derived from actinomycetes is lacking. The discovery of secretory proteins with induced resistance has opened up a broad prospect for diversified use of protein-induced disease resistance to control plant diseases.

Hydrogen peroxide (H_2_O_2_), one of the most stable ROS, has been identified as a key signaling regulator of plant physiological processes such as disease resistance (Petrov and Van, 2012). The biological function of H_2_O_2_ is concentration-dependent, and high concentrations of H_2_O_2_ can cause cell death (Mhamdi et al., 2012). ROS are believed to be a signaling molecule for plant defense and interact with other signaling networks in plants (Apel and Hirt, 2004; Miller et al., 2008; Mittler et al., 2011). ROS accumulation is controlled by enzymes that detoxify ROS, such as superoxide dismutase (SOD), catalase (CAT), ascorbate peroxidase (POD), and glutathione reductase (Apel and Hirt, 2004). CATs are peroxisomal proteins that scavenger ROS by converting H_2_O_2_ to water and oxygen in nearly all living organisms (Inaba et al., 2011). *Arabidopsis thaliana* harbors three CAT genes, CAT1, CAT2, and CAT3 (Frugoli et al., 1996). CAT1 has a key role in removing H_2_O_2_ that is produced under a diverse range of environmental stresses. CAT2 and CAT3 are highly expressed and localized in the peroxisome. All these CAT genes are important players in detoxifying H_2_O_2_ that controls plants’ ROS homeostasis (Du et al., 2008). CATs also play important roles in plant immunity. For example, the cucumber mosaic virus (CMV) 2b protein, the viral RNA silencing suppressor, directly interferes with plant CATs to induce programmed cell death (PCD) via the degradation of CAT3, which appears to facilitate CMV infection (Murota et al., 2017). Two effectors, PsCRN63 and PsCRN115 from the oomycete *Phytophthorasojae*, regulate plant cell death and H_2_O_2_ homeostasis through a direct interaction with CAT to deal with host immune responses (Zhang et al., 2014, 2015). Some proteins in plant cells, such as lesion stimulating disease 1 (LSD1) (Li et al., 2013) and no catalase activity 1 (NCA1) (Li et al., 2015), also regulate the HR in multiple stresses by interacting with CATs.

*S*train Hhs.015, a PGPR strain well-studied for biocontrol in crop cultivation, was first isolated from cucumber roots and classified in the genus *Saccharothrix* (Yan et al., 2012). According to the genome-wide sequencing results of strain Hhs.015, we analyzed its secreted protein genes using several software of SignalPv4.1, TMHMMv2.0, DAS-TMfilter, HMMTOP, Prosite Scan, PSORT and big-PI predictor and selected some of the function-unknown putative protein genes to investigate their functions after heterologous expression. According to the disease resistance to apple canker and defense-related enzyme activity, a candidate elicitor protein BAR11 was obtained. In this study, we demonstrated that the recombinant protein BAR11 triggered early signaling events of plant defense responses in *A. thaliana* and ISR against infections by *Pseudomonas syringae* pv. *tomato* (*Pst*) DC3000.

## Materials and Methods

### Plants, Strains, and Growth Conditions

*Nicotiana benthamiana* plants were cultivated in a phytotron at 24°C with a 12-h day/night cycle. *Arabidopsis* seeds were sown in a suitable controlled plant growth condition at 22°C with a 12-h day/night cycle at 75% relative humidity (Niu et al., 2011).

*Pseudomonas syringae* pv. *tomato* (*Pst*) DC3000 was cultured in liquid Kings’ B medium containing 50 mg of rifampicin per liter at 28°C overnight as previously described (Niu et al., 2011). Culture cells were harvested and the final concentration of cell suspensions was adjusted to 5 × 10^8^ CFU/mL using 10 mM MgSO_4_ containing 0.01% (vol/vol) surfactant Silwet L-77 (Sigma, St. Louis, MO, United States).

*Valsa mali* strain 03-8 was grown on potato dextrose agar plates and incubated at 25°C in the dark for 3 days before inoculation onto apple leaves.

### Prokaryotic Expression and Purification of Protein Elicitor BAR11

The sequence for *BAR11* without the signal peptide was inserted into the pET28a vector (Novagen, United States) upstream and downstream of two 6×His tags. The final plasmid was then introduced into *Escherichia coli* BL21 (DE3) cells to express the BAR11-His recombinant protein. The *E. coli* BL21 cells were subsequently grown in LB-medium containing 50 mg of kanamycin per liter at 37°C to an optical density at 600 nm (OD_600_) of 0.5–0.6, and then induced with 0.1 mM isopropyl b-D-1-thiogalactopyranoside (IPTG) (Sigma, United States) for 18–20 h at 16°C, from which the protein BAR11-His was extracted as follows. The induced culture cells were harvested by centrifugation (5,000 ×*g*, 20 min). The insoluble fraction was resuspended in phosphate-buffered saline (pH7.4) and disrupted using an ultrasonic disruptor (Scientz, China). After centrifugation (18,000 ×*g*, 4°C, 30 min), the supernatant containing recombinant protein was gathered into a new pellet and filtered through a 0.22-μm filter (Millipore, China). The filtrate was then purified via a HisTrapTM HP column (GE Healthcare, United States) following the manufacturer’s instructions. Further purification of the eluted protein was performed via dialysis desalination and freeze-drying. The protein purity and molecular weight were determined by sodium dodecyl sulfate-polyacrylamide gel electrophoresis (SDS-PAGE) and staining with Coomassie Brilliant Blue G250. The protein was then used for biological activity assay.

### Bioassay for BAR11-Induced Disease Resistance

The disease control ability of BAR11-treated plants was determined by supervising for disease symptoms after pathogen inoculation with protein elicitors at a concentration of 20 μM. This was determined by detached soaking ‘Fuji’ apple leaves for 10 min with the elicitor. For the negative control, sterile water was used in the same way. The apple leaves were inoculated with *V. mali* at 24 h after BAR11 treatment, and the inoculation assay was conducted as described below (Li et al., 2015, 2016).

Four-week-old *A. thaliana* leaves were treated with 5 mL of 20 μM recombinant BAR11, and sterile water was used as the control. Three days later, systemic leaves were sprayed with *Pst* DC3000 cell suspension in 10 mM MgSO_4_ containing surfactant Silwet L-77 (OD_600_ = 0.1). Inoculated plants were maintained in a growth chamber (Percival AR800, United States) at 22°C with 70% relative humidity and a 16-h day/8-h night cycle (Liu et al., 2016).

### Detection of H_2_O_2_ Production and Callose Deposition in *A. thaliana*

The production of H_2_O_2_ in the *A. thaliana* leaves was observed as previously described (Niu et al., 2011) without any change in our study. The *A. thaliana* leaves were treated with 20 μM BAR11, and sterile water was used the control. The leaf sections of 12 h post-treatment (hpt) were harvest for DAB-staining. The final leaves were then placed in a destaining solution bath (0.15% trichloroacetic acid w/v dissolved in 3:1 ethanol mixed with chloroform solution) to clear the chlorophyll, and then leaf segments were preserved in the saturated chloral hydrate to be transparent for 2–3 days. H_2_O_2_ production in the *A. thaliana* leaves was then examined under a light microscope (Olympus BX51, Japan). To determine callose deposition, *A. thaliana* leaves were sampled at 12 hpt and stained by the aniline blue method as described previously (Niu et al., 2011) without any modification in our study. These experiments were performed three times in the same way.

### RT-PCR Analysis of Gene Expression

The relative expression levels of pathogenesis-related proteins (*PR1, PR2*, and *PR5*), radical-responsive glutathione transferase (*GST1*), lipoxygenase 1 (*LOX1*), lipoxygenase 2 (*LOX2*), plant defensin gene 1.2 (*PDF1.2*), and non-expressor of pathogenesis related 1 (*NPR1*) were analyzed in plants. Total RNA was extracted from frozen *A. thaliana* leaf samples using a quick RNA isolation kit (Huayueyang, Beijing, China). Using the oligo dT primers, the first-strand cDNA was synthesized in 20-μL reactions containing 1 μg total RNA using the RevertAid first strand cDNA synthesis kit (Thermo, United States). Primers specific to *PR1, PR2, PR5, GST1, LOX1, LOX2, PDF1.2, NPR1*, and *EF-1a* were designed using the Primer Quest Tool by IDT^1^. Twenty-five cycles of independent PCR were performed using 1-μL cDNA samples and gene-specific primers. *EF-1a*, a constitutively expressed gene, was used as an endogenous control in the RT-PCR examination.

### Localization and Bimolecular Fluorescence Complementation Assay

Agroinfiltration was carried out following Goto et al. (2007) with a slight modification. Briefly, a cell suspension of pre-cultured *Agrobacterium tumefaciens* strain GV3101 carrying an expression plasmid (pCAMBIA-1302:BAR11:GFP) was adjusted to OD_600_ = 0.4 and infiltrated into the upper leaves of 4-week-old *N. benthamiana* plants using a 1-mL syringe. *A. tumefaciens* cells carrying the *bar* gene (pCAMBIA-1302:*bar*) were subsequently infiltrated into the same site. Control plants were infiltrated with *A. tumefaciens* carrying an empty pCAMBIA-1302 vector. The *A. tumefaciens* leaves were then shredded 2–3 days post-injection (dpi), and a laser confocal microscope (Olympus BX-51) with an excitation wavelength of 485 nm was then used to observe for the localization of green fluorescent protein (GFP). Furthermore, 0.8 M of sorbitol was used to treat the tobacco cells for 15 min for analyzing the fluorescence distribution.

### Co-immunoprecipitation Experiments and Western Blot Analysis

For immunoprecipitation experiments, total protein was extracted from the inoculated *N. benthamiana* leaves at 2 dpi with the 1302:BAR11:GFP or 1302:AtCAT (1, 2, 3):HA constructs, and subjected to immunoprecipitation via GFP-Trap^®^_A per the manufacturer’s protocol. GFP-trap beads (20 μL; Chromotek) were used following the standard operating protocols to immunoprecipitate GFP-bar::AtCAT (1, 2, 3)-HA from protein extracts (Lyzenga et al., 2013).

Filtrated leaves from transiently expressed leaves of *N. benthamiana* were harvested and frozen in liquid nitrogen. Samples frozen with liquid nitrogen were lysed in 200 μL Lysis Buffer (50 mM Tris-HCl, pH 7.4, 150 mM NaCl, 1 mM EDTA, 5 mM DTT, 0.1% Triton X-100, 1× protease inhibitor cocktail, and 1 mM PMSF) in an ice-bath for 30 min and vortexed every 10 min, and then 100 μL of loading buffer was added (Hong et al., 2017). The extracts were boiled for 10 min and then centrifuged at 12000 ×*g* for 10 min at 4°C. The supernatant (20 μL) was separated on a 10% SDS-PAGE gel, followed by a transfer onto PVDF membrane via wet electroblotting. For the co-immunoprecipitation assay, detection of the GFP or HA tags in transiently expressed *N. benthamiana* leaves was performed using a mouse monoclonal GFP or HA antibody (Sigma-Aldrich) for 12 h at 4°C, respectively, followed by alkaline phosphatase-conjugated goat anti-mouse IgG secondary antibody for 1 h at 25°C. The alkaline phosphatase chromogen kit (Thermo, United States) was used to detect the secondary antibody.

### Statistical Analysis

All data were analyzed separately using the SPSS 16.0 software (SPSS Inc., United States). Significant differences between mean values were determined using the student’s *t*-test (*P* < 0.05).

## Results

### Characterization of BAR11

**FIGURE 1:**
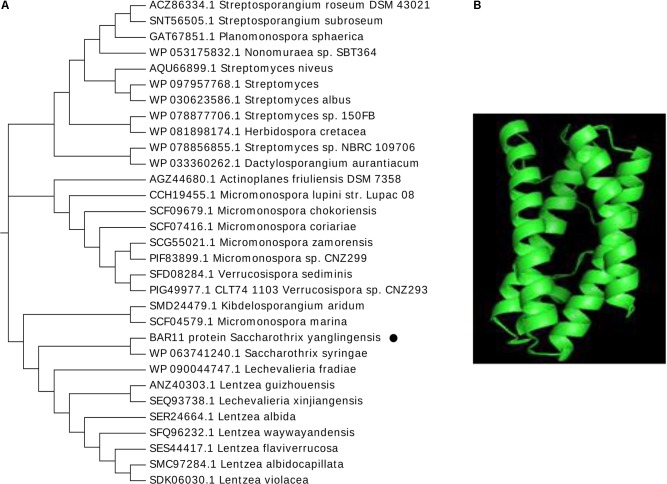
Characterization of BAR11 from *Saccharothrix yanglingensis* Hhs.015. **(A)** Maximum likelihood phylogenetic tree of BAR11. **(B)** Tertiary structure prediction.

BAR11 is a hypothetical protein secreted by *Saccharothrix yanglingensis* strain Hhs.015. The results showed that the molecular weight of the BAR11 peptide is 27900 Da, the number of negatively charged residues is 21, and the number of positive residues is 25. In addition, the peptide also consists of 23.1% alanine, 13.6% leucine, and 10.1% glycine. A Secondary Structure Prediction Server analysis for the secondary structure of BAR11 indicated that the peptide chain has four α-helices with no β-folding and irregular curl. The predicted results for the transmembrane domain showed that there is a transmembrane domain in the N-terminal region, which has a distinct hydrophilic and hydrophobic region and a distinct hydrophilic region at position 120–125. Furthermore, the N-terminal, C-terminal, and central regions of the sequence have a hydrophobic region. A BLAST comparison showed that the BAR11 nucleotide sequence (Accession number: MG586232) shares the highest sequence similarity to proteins of several beneficial microorganisms and contains the conserved domain of DUF305 (**Figure 1A**). The three-dimensional structure of the protein DUF305 secreted by *Streptomyces coelicolor* (2qf9. 1.A) was used as a template to model the three-dimensional structure of the BAR11 sequence. The structure is mainly composed of an α-helix, and the same result was obtained with the Phyre^2^ Prediction Server (reliability, 100%; coverage, 73%; **Figure 1B**).

### BAR11 Enhances Disease Resistance in Plants

**FIGURE 2:**
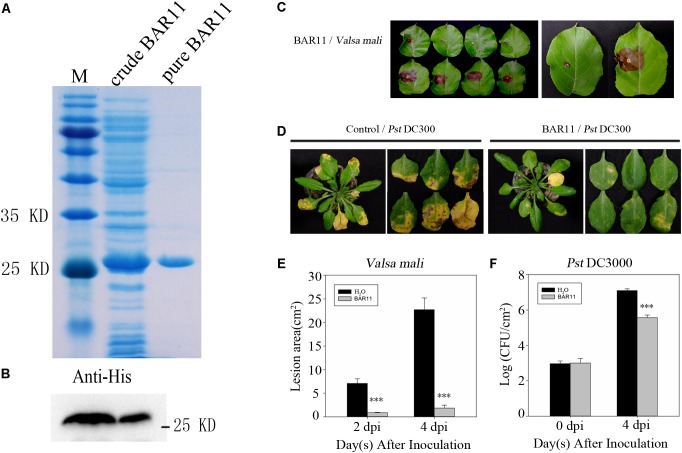
BAR11 induces effective plant defense against pathogen infection. **(A)** Purification and characterization of recombinant BAR11. Lane M, protein molecular weight marker; Lane 1, total *Escherichia coli* expressed proteins; Lane 2, purified His-tagged BAR11. **(B)** The presence of BAR11 was confirmed by immunoblotting using anti-His antibodies. **(C)** Plants were syringe-infiltrated with BAR11 protein. Twenty-four hours later, apple leaves were challenged by *Valsa mali*. BAR11 reduced apple Valsa canker lesion development. **(D)** Three days later, *Arabidopsis thaliana* leaves were challenged by syringe-infiltration with 5 × 10^5^ CFU/mL *Pseudomonas syringae* pv. *tomato* (Pst) DC3000. Symptoms were observed 4 days after infection with Pst DC3000 as well as BAR11-induced resistance against DC3000. **(E)** The lesion areas are expressed as mean ± standard deviation from three independent experiments. **(F)** Pst DC3000 density in the leaves of *A. thaliana* plants. Plants were inoculated with Pst DC3000 at 3 days post-treatment (dpt), and then leaves were harvested at 4 days post-inoculation (dpi). Bars represent average numbers of CFU per gram of leaf fresh weight. Statistical analyses were performed using Student’s *t*-test, and asterisks indicate significant differences between BAR11 and Sterile water treatments (^∗∗∗^*P* < 0.001).

Functional analysis of BAR11 was investigated via heterologous expression in *E. coli* to produce enough protein. The crude extracts were applied onto a HisTrapTM HP column (GE Healthcare), eluted with 50–200 mM imidazole, and then purified by dialysis desalination and freeze-drying (Bu et al., 2014). Finally, the recombinant protein showed a single band with the relative molecular mass of 27 kDa by SDS-PAGE analysis (**Figure 2A**). The protein concentration was 10 μM as determined using a BCA Protein Assay Kit (PIERCE, United States), followed by a transfer onto a PVDF membrane via wet electroblotting. His-BAR11 was then subjected to Western blot analysis using a mouse monoclonal His antibody (**Figure 2B**).

In order to investigate whether BAR11 can enhance plant disease resistance, apple leaves were inoculated with the pathogen *V. mali* at 24 hpt after treatment with BAR11 (1 mg/mL) or sterile water. Compared with sterile water-treated control leaves, BAR11-treated leaves showed noticeable weaker disease symptoms (**Figures 2C,E**). At 4 dpi, pathogen density in *A. thaliana* leaves which were pretreated with BAR11 was led to a significant reduction (*P* < 0.05) in pathogen density in *A. thaliana* leaves. *A. thaliana* bottom leaves were infiltrated with BAR11 or sterile water. Three days after the initial treatment, *A. thaliana* upper leaves (non-treated with BAR11) on the same plant were challenged with a virulent strain of DC3000. Plants with different pre-treatments and DC3000 infections were examined 4 dpi. We observed that BAR11 induced effective SAR against *Pst* DC3000 infection (**Figures 2D,F**). These results indicate that BAR11 significantly increased plant defense levels against the pathogen.

### H_2_O_2_ Accumulation and Callose Deposition Induced by BAR11 in *Arabidopsis*

**FIGURE 3:**
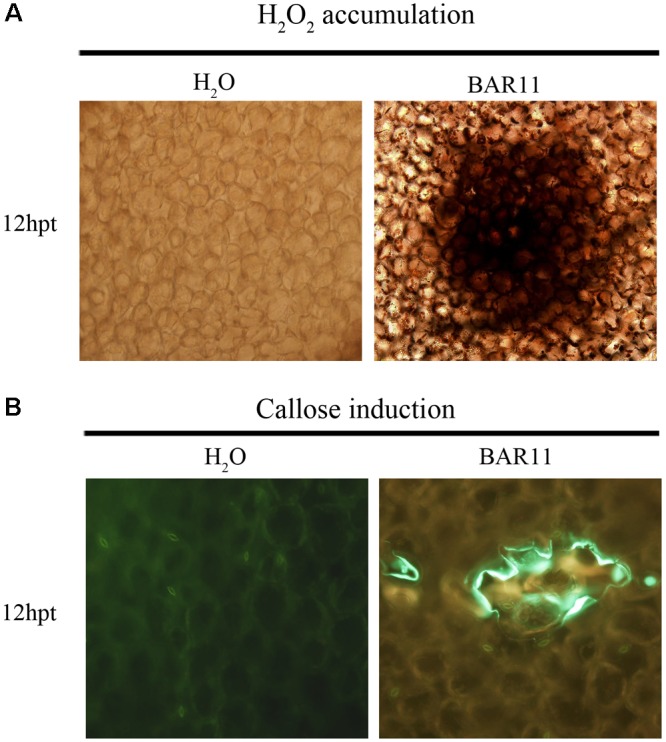
BAR11 promotes H_2_O_2_ accumulation and callose deposition. **(A)** H_2_O_2_ production in *Arabidopsis* leaves. DAB staining was performed at 12 h post-treatment (hpt) with protein BAR11. **(B)** Callose deposition in *A. thaliana* leaves. Aniline blue staining was performed at 12 hpt with BAR11 protein.

Systemic acquired resistance is usually associated with the occurrence of early cell defense reactions, such as rapid explosion of reactive oxygen and accumulation of callose. To investigate whether BAR11-induced disease resistance is associated with primed defense responses in leaves, we examined H_2_O_2_ accumulation and callose deposition in *A. thaliana*. H_2_O_2_ accumulation and callose deposition induced by BAR11-treatment and H_2_O control were detected at 12 hpt in the *A. thaliana* leaves of plants (**Figure 3**). H_2_O_2_ was assayed using DAB and significant brown precipitates were observed in recombinant BAR11-treated *A. thaliana* leaves. Callose deposition in the *A. thaliana* leaves was visualized with ultraviolet excitation.

### BAR11 Treatment Induces Defense-Related Gene Expression

**FIGURE 4:**
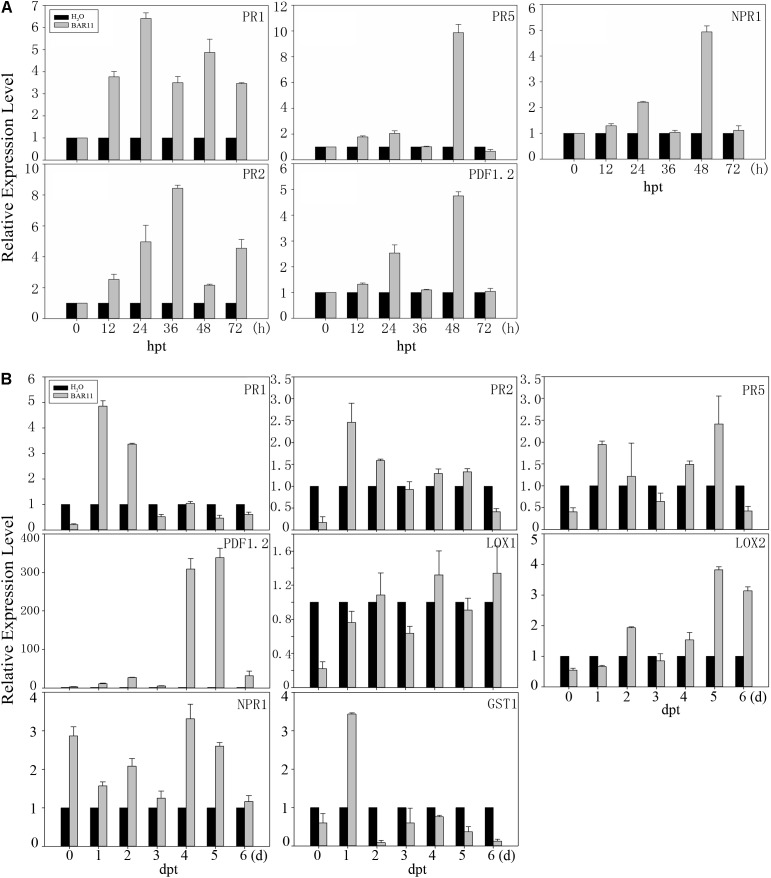
Expression analysis of defense-related genes in apple and *A. thaliana* leaves after BAR11 treatment. **(A)** The apple leaf samples were harvested from systemic leaves at the indicated times, and qRT-PCR was performed to demonstrate the relative expression levels of genes encoding PR1, PR2, PR5, NPR1, and PDF1.2. **(B)** The *A. thaliana* leaf samples were harvested from systemic leaves at the indicated times, and qRT-PCR was performed to show the relative expression levels of genes encoding PR1, PR2, PR5, NPR1, PDF1.2, LOX1, LOX2, and GST1. Samples were normalized against EF-1a, and expression levels are represented as fold changes in relation to the control.

To further investigate the resistance mechanisms of recombinant BAR11-treated plants, we soaked apple leaves and sprayed 4-week-old *A. thaliana* leaves with recombinant BAR11 protein and then detected the expression levels of several plant defense-related genes. The pathogenesis-related proteins PR1, PR2, and PR5 are all markers of the SA-dependent defense pathway (Uknes et al., 1992). The qRT-PCR results for the apple leaves demonstrated that these pathogenesis-related proteins were significantly upregulated in response to recombinant BAR11 (**Figure 4A**). The *PR1* expression reached a peak by 6.4-fold at 24 hpt after BAR11 treatment. Expression of PR2 continuously increased by 8.4-fold at 36 hpt. PR5 was significantly upregulated by approximately 10-fold at 48 hpt. The levels of these genes then decreased, but were still upregulated when compared to that in the control. Meanwhile, recombinant BAR11 treatment triggered the expression of PDF1.2 (**Figure 4A**), which is a crucial JA-responsive protein. The BAR11-induced PDF1.2 reached 2.4-fold at 24 hpt after the recombinant BAR11 treatment, and was subsequently upregulated by 4.8-fold at 48 hpt. Furthermore, *NPR1* was upregulated by 4.9-fold (**Figure 4A**). The qRT-PCR results for the *A. thaliana* leaves demonstrated that the expression of pathogenesis-related proteins PR1, PR2, and PR5 was significantly upregulated in response to recombinant BAR11 (**Figure 4B**). The expression levels of the PR1, PR2, and PR5 gene were significantly upregulated at 1 and 2 dpt, and the maximum level of the gene increased by 4.9-fold at 1 dpt. Meanwhile, recombinant BAR11 triggered LOX2 expression and PDF1.2 upregulation (**Figure 4B**); PDF1.2 expression was increased by 310-fold at 4 dpt and subsequently upregulated by 338-fold at 5 dpt. All these are JA-responsive marker genes. *NPR1* and *GST1* were also upregulated (**Figure 4B**). The present results indicate that the BAR11-induced host plants’ resistance to pathogens may affect both SA and JA/ET signaling pathways. Furthermore, qRT-PCR demonstrated notable induction of these marker genes, suggesting an induction of SA and JA signaling in BAR11-treated plants.

### BAR11 Associates With CATs From *N. benthamiana* and *Arabidopsis*

**FIGURE 5:**
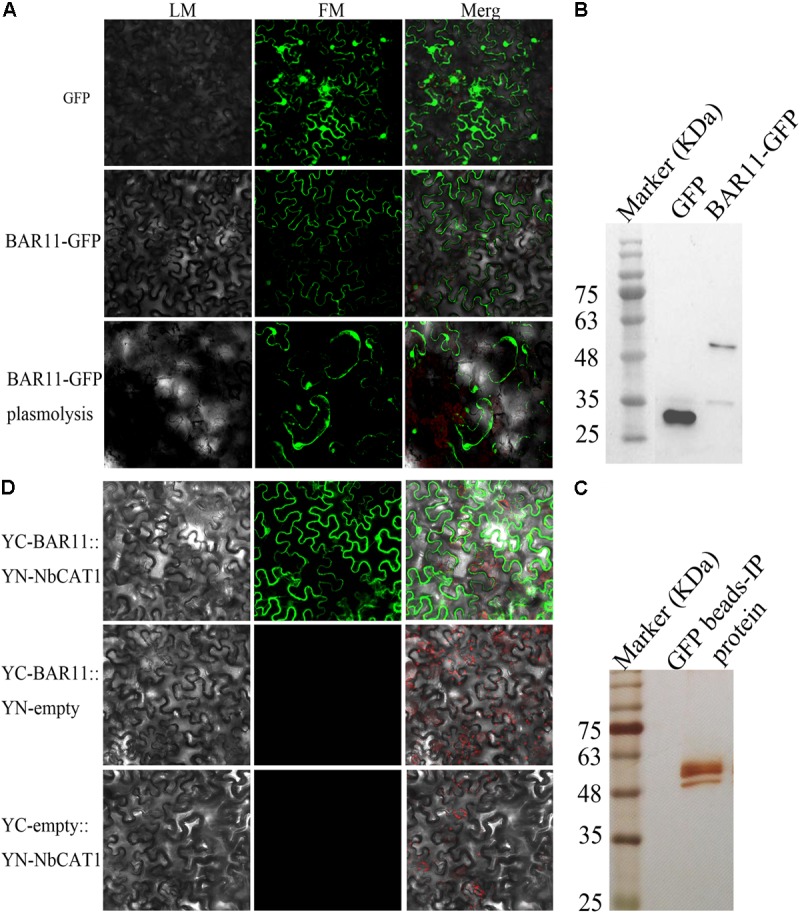
BAR11 associates with CATs from *Nicotiana benthamiana* and *A. thaliana*. **(A)** Sub-cellular localization of BAR11. The pCAMBIA-1302-bar11-egfp construct transfected into *N. benthamiana* leaf lower epidermis. **(B)** The presence of the BAR11-GFP or GFP protein was confirmed by immunoblotting using anti-GFP antibodies. **(C)** Identification of BAR11-interacting protein(s) by co-immunoprecipitation assay. Total protein isolated from *A. thaliana* Col-0 plants treated 2 days post-infiltration was used for co-immunoprecipitation assays. Samples were separated by SDS-PAGE and visualized by silver staining. The protein band (indicated by the red arrow) was excised and identified by mass spectrometry. Relevant molecular mass markers are shown on the left in kDa. **(D)** Bimolecular fluorescence complementation detection of BAR11-NbCAT1 interactions in sub-cellular locations.

The cellular localization of BAR11 was determined by generating a fusion with enhanced green fluorescent protein (eGFP). To this end, we fused *bar11* without the signal peptide sequence to *egfp*, to produce the pCAMBIA-1302-bar11*-egfp* construct. Then the *A. tumefaciens* strain carrying the pCAMBIA-1302-bar11*-egfp* construct was infiltrated into *N. benthamiana* leaves, and GFP fluorescent signals were observed in the nucleus and membrane. After 15-min treatment with 0.8 M sorbitol, the cells were clearly separated from the cell wall and the fluorescence was localized in the cell membrane and nucleus (**Figure 5A**). Isolated proteins were used for Western blot analysis with GFP antibodies (**Figure 5B**).

To identify potential protein targets in *N. benthamiana*, *in planta* co-immunoprecipitation with GFP-Trap^®^_A beads was conducted, followed by liquid chromatography–tandem mass spectrometry (**Figure 5C**). The results showed that there were additional candidate target proteins, and combined with the pre-active oxygen accumulation phenotype observed, which suggests that CAT may be the target of BAR11 interaction. Then the interaction between the candidate and BAR11 was examined via bimolecular fluorescence complementation. The *A. tumefaciens* strain carrying the BAR11-YC and NbCAT1-YN constructs were co-infiltrated into *N. benthamiana* leaves, and the results showed that BAR11 could interact with NbCAT1 (**Figure 5D**).

### BAR11 Affects Plant Immunity by Interacting With AtCATs

**FIGURE 6:**
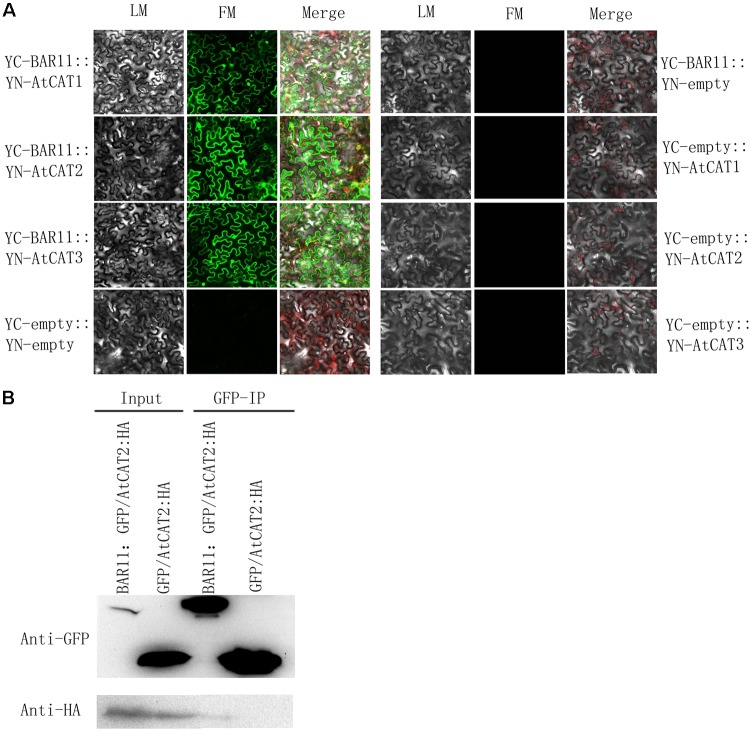
BAR11 interacts with plant catalases. **(A)** Bimolecular fluorescence complementation detection of BAR11-AtCATs interactions in sub-cellular locations. **(B)** Analyses of BAR11 and AtCAT2 proteins via Western blot analysis. Co-immunoprecipitation of BAR11 with catalase 2 encoded by *A. thaliana*. BAR11:GFP and AtCAT2:HA were transiently co-expressed in *N. benthamiana*. The co-immunoprecipitation was performed using an anti-HA antibody, and the isolated protein was analyzed by immunoblotting using an anti-GFP antibody to detect BAR11 and an anti-HA antibody to detect catalase 2.

The *A. thaliana* genome contains a small group of homologous genes belonging to the catalase family, including CAT1, CAT2, and CAT3 (Frugoli et al., 1996). In order to further clarify whether BAR11 interacts with AtCATs (1, 2, 3), BAR11 and AtCATs (1, 2, 3) on the YC-1301 and YN-1301 were constructed, and the recombinant vectors BAR11-YC and AtCATs (1, 2, 3)-YN were then obtained. To determine the subcellular site(s) for interaction of the elicitor BAR11 interacts with plant catalases, BAR11-YC and AtCATs (1, 2, 3)-YN fusions were transiently co-expressed via agroinfiltration in *N. benthamiana* leaves, with the empty vectors YN-1301 and YC-1301 as negative controls. Yellow fluorescence signal was observed under laser confocal microscopy. Co-expression of BAR11-YC with AtCATs (1, 2, 3)-YN (**Figure 6A**) in tobacco leaf epidermal cells resulted in a fluorescent signal in the nucleus and cytoplasm. In contrast, transient expression of the empty vectors YN-1301 and YC-1301 did not trigger YFP. Taken together, the results suggest that there was an interaction between BAR11 and AtCATs (1, 2, 3).

In the *A. thaliana* vegetative tissues, CAT2 and CAT3 play key roles to represent major catalase activity (Frugoli et al., 1996; Mhamdi et al., 2010). As a result, here, possible interactions of BAR11 with CAT2 in plants were tested. The *A. tumefaciens* strain carrying the GFP:BAR11 construct was co-infiltrated with that containing the AtCAT2:HA construct into *N. benthamiana* leaves, and total proteins from the treated *N. benthamiana* leaves at 2 dpi were extracted for follow-up experiments. We used the GFP-trap beads (20 μL; Chromotek) to immunoprecipitate GFP-BAR11 from total protein extracts. Isolated proteins were then analyzed by Western blot with GFP or HA antibodies (**Figure 6B**). The results demonstrated that the elicitor BAR11 interacted with AtCAT2 and then interfered with the plant’s H_2_O_2_ levels, thus influencing the defense pathways and improving plants resistance against pathogens.

## Discussion

Induced resistance is one of the most important mechanisms of disease resistance. It is a non-traditional, eco-friendly method for plant protection. Eventually, enhanced resistance occurs in plants due to the induction of defense responses. Related research mainly focuses on the protective responses of the cell wall, accumulation of plant defensin proteins, activity of the related enzymes, synthesis of new proteins, and molecular signaling and transmission pathways (Reuber et al., 1998). Microbial elicitors capable of triggering defense responses both in host and non-host plants are general elicitors (Kruger et al., 2003). Elicitors are compounds belonging to a wide range of different classes without any common chemical structure, including peptides, proteins, oligosaccharides, glycoproteins, and lipids (Montesano et al., 2003). Protein elicitor-induced plant resistance has attracted considerable attention and research effort in recent years for alternative, novel, and eco-friendly plant protection methods (Mishra et al., 2012).

Elicitors are an attractive potential alternative to fungicides due to the fact that the former can trigger plant defense responses (Wiesel et al., 2014). Researchers have described that there are numerous elicitors of different nature protecting plants against pathogens. The novel elicitor SsCut protein can activate defense responses by inducing a typical HR response in tobacco leaves and production of multiple signaling molecules and secondary metabolites related to plant resistance (Zhang et al., 2014). Wang et al. (2016) reported that a novel elicitor protein from *B. amyloliquefaciens* strain NC6 secreted a novel elicitor, designated PeBA1; this protein can trigger a series of defense HR in tobacco leaves, activate defense-related early events to, upregulate defense-related genes, and promote accumulation of antimicrobial compounds that can enhance tobacco disease resistance to tobacco mosaic virus (TMV) and *Botrytis cinerea*. Our previous studies have shown that *S. yanglingensis* strain Hhs.015 was able to colonize in apple tissue culture seedlings and trigger ISR by improving resistance-related enzyme activity (Fan et al., 2016). Based on genomic sequencing, the elicitor protein BAR11 was identified. In this study, it was found that BAR11 can trigger an immune response to enhance resistance in plants against *V. mali* and *Pst* DC3000. Beneficial microorganisms can induce systemic defense reaction, and the reaction of these plant hormones SA, JA, and the ethylene (ET) plays an important role in the control of the signaling network (Hammond-Kosack and Parker, 2003). Some evidences have shown that depending on the different pathogen, the SA, JA, and ET pathways crosstalk regulates plant defense responses (Koornneef and Pieterse, 2008).

Analysis via the Tertiary Structure Prediction Server predicted that the tertiary structure is mainly composed of four simple and stable α-helices. Moreover, the BAR11 protein sequence has low similarity compared with other protein sequences in the database. According to BLAST result, the closely related sequences belong to actinomycetes such as *Saccharothrix syringae*, *Streptomyces* sp., and *Lentzea* (Supplementary Figure S3).

BAR11 does not cause an inhibition effect of *V. mali in vitro*; however, it can induce a defense response in apple leaves against *V. mali* at 24 hpi and can improve the activity of defense-related enzymes. Moreover, prophylactic application of BAR11 on *A. thaliana* leaves at 3 dpi leads to a significant inhibition of *Pst* DC3000. These results suggest that the induced protection could be attributable to plant defenses, but not direct toxicity to the pathogens. Other elicitors obtained from other sources also have these features. For example, Harpin, which has been identified in numerous pathogens, enhances host resistance against different pathogens, and is involved in the ethylene (ET) and JA signaling pathways (Shao et al., 2008). Fungal chitosan could enhance the immunity of plants, enhance orchid production and induce differentiation in orchid plant tissue (Nge et al., 2006; Uthairatanakij et al., 2007). The novel elicitor AsES triggers a dose- and time-dependent defense response to *B. cinereal*, and SA-, JA-, and ET-induced signaling pathways play a fundamental role in activating AsES-dependent responses (Hael-Conrad et al., 2015). It was found that BAR11 induced activities of Phenylalanine ammonia lyase (PAL), CAT, POD, and SOD and suppressed *Pst* DC3000 infection (Supplementary Figure S1) (Weydert and Cullen, 2010). Following stimulation by BAR11, the SA and JA signaling pathways may be activated. These results suggest that the BAR11-treatment enhanced plants’ disease resistance against pathogens.

Reactive oxygen species play dual roles or more in plant cells, depending on their intracellular concentrations (Kotchoni and Gachomo, 2006). At higher concentrations, ROS cause extensive cell injury or death (Li et al., 2013), whereas at biologically balanced levels, ROS act as signaling molecules to induce defense responses against pathogens. ROS production, including H_2_O_2_ and O_2_^-^, results mainly from the activity of SOD and NADPH oxidases, respectively. Plant catalase, a H_2_O_2_-decomposing peroxisomal enzyme, plays an essential role in maintenance of H_2_O_2_ homeostasis and regulation of PCD in plant cells (Mhamdi et al., 2010). Interestingly, BAR11 can induce H_2_O_2_ accumulation and callose deposition in *A. thaliana* leaves. H_2_O_2_ is an important signaling molecule for programmed cell death, and we found that BAR11 cannot induce localized hypersensitive cell death (Supplementary Figure S2). However, it can induce hydrogen peroxide accumulation, although the exact mechanism is not clear. Based on the above enzyme activity results, we hypothesized that BAR11 activates both SOD and CAT activity, breaks down the original H_2_O_2_ level, and maintains a high level of H_2_O_2_ in the treated plants compared with the control. BAR11 cannot induce localized hypersensitive cell death (HR or PCD), but can increase plant resistance.

Based on previous studies, these results prompted us to initiate a search for targets of BAR11 that interact with tobacco via GFP-Trap^®^_A beads immunoprecipitation assays in an attempt to identify tobacco proteins able to bind to BAR11. Mass spectrometry sequencing results showed that there are candidate target catalases produced in the peroxisome, which are the undisputed scavengers of H_2_O_2_. It can effectively maintain H_2_O_2_ levels and play an important role in the normal metabolism of plants, aging, and stress response. Previous studies showed that several plant proteins, including NDK1, SOS2, NCA1, and LSD, can interact with CATs and increase CAT enzyme activities, leading to decreased H_2_O_2_ concentrations and inhibited PCD in plant cells (Fukamatsu et al., 2003; Verslues et al., 2007; Li et al., 2013, 2015). Recently, two effector proteins, PsCRN63 and PsCRN115, have been identified from the oomycete *Phytophthora sojae*, which can directly interact with plant CAT and is localized in the nucleus. The former can induce the latter, while the latter inhibits the host PCD (Zhang et al., 2014). RipAK interferes with ROS-mediated signaling to inhibit CATs, resulting in the suppression of immune responses at early stages of plant immunity (Sun et al., 2017).

## Conclusion

BAR11 can enhance plant disease resistance to *Pst* DC3000 and *V. mali* by inducing accumulation of early defense events and defense-related gene up-regulation. Our results helps elucidate the mechanisms of BAR11-triggered systemic resistance in plants. BAR11 interacts directly with plant catalases by converting H_2_O_2_ to H_2_O and O_2_; this indicates that BAR11 may modulate plant immune responses by perturbing H_2_O_2_ homeostasis. At higher concentrations, H_2_O_2_ can cause extensive cell injury or death. However, BAR11 induces higher levels of H_2_O_2_ and cannot cause PCD. To our knowledge, this is the first study to show that a novel elicitor from actinomycete can regulate plant immunity and interact with plant catalases. Therefore, the elicitor BAR11 may be useful as an effective alternative for inducing plant defenses against pathogens. Our future research will be aimed at illuminating the potential host-pathogen-elicitor interaction mechanism. Further studies are needed to elucidate the exact mechanisms underlying BAR11-induced priming of plant defense responses. These finding will contribute to a better understanding of the biological functions and molecular mechanisms of BAR11 in modulation of plant defense mechanisms and lay the foundation for future studies.

## Author Contributions

YZ, XY, and LH were responsible for the experimental design, and provided guidance on the whole study. YZ and HG carried out experiments. YZ wrote the manuscript and XY and LH further revised it. FZ analyzed some experimental results.

## Conflict of Interest Statement

The authors declare that the research was conducted in the absence of any commercial or financial relationships that could be construed as a potential conflict of interest.

## Footnotes

1 http://sg.idtdna.com/Primerquest/Home/Index

## Supplementary Material

**FIGURE S1 |** Effect of BAR11 treatment on CAT, PAL, SOD, and POD activities. *A. thaliana* leaves were collected from BAR11-treated plants and sterile water controls at 0, 3, 6 ,12, and 24 h after treatment. The effect of BAR11 treatment on CAT, PAL, SOD, and POD activities in *A. thaliana* leaves was determined by Hano et al. (2008), Weydert and Cullen (2010) methods. Data are expressed as mean of triplicate samples and standard deviation.

**FIGURE S2 |** Symptoms of transient expression of BAR11 in *N. benthamiana* leaves after agro-infiltration. Leaves were infiltrated with *A. tumefaciens* containing a PVX vector, carrying the BAR11 gene, or carrying the Bax gene, and H_2_O with in the regions indicated by the dashed lines.

**FIGURE S3 |** Sequence alignment of the amino acid sequence of BAR11. Identical amino acid residues are shaded in black and similar residues are shaded in gray.
