# Supplementary figures and images for: A Novel Protein Elicitor BAR11 From Saccharothrix yanglingensis Hhs.015 Improves Plant Resistance to Pathogens and Interacts With Catalases as Targets
Source: Front Microbiol. 2018 Apr 9;9:700. doi: 10.3389/fmicb.2018.00700 (PMC5900052; doi:10.3389/fmicb.2018.00700)

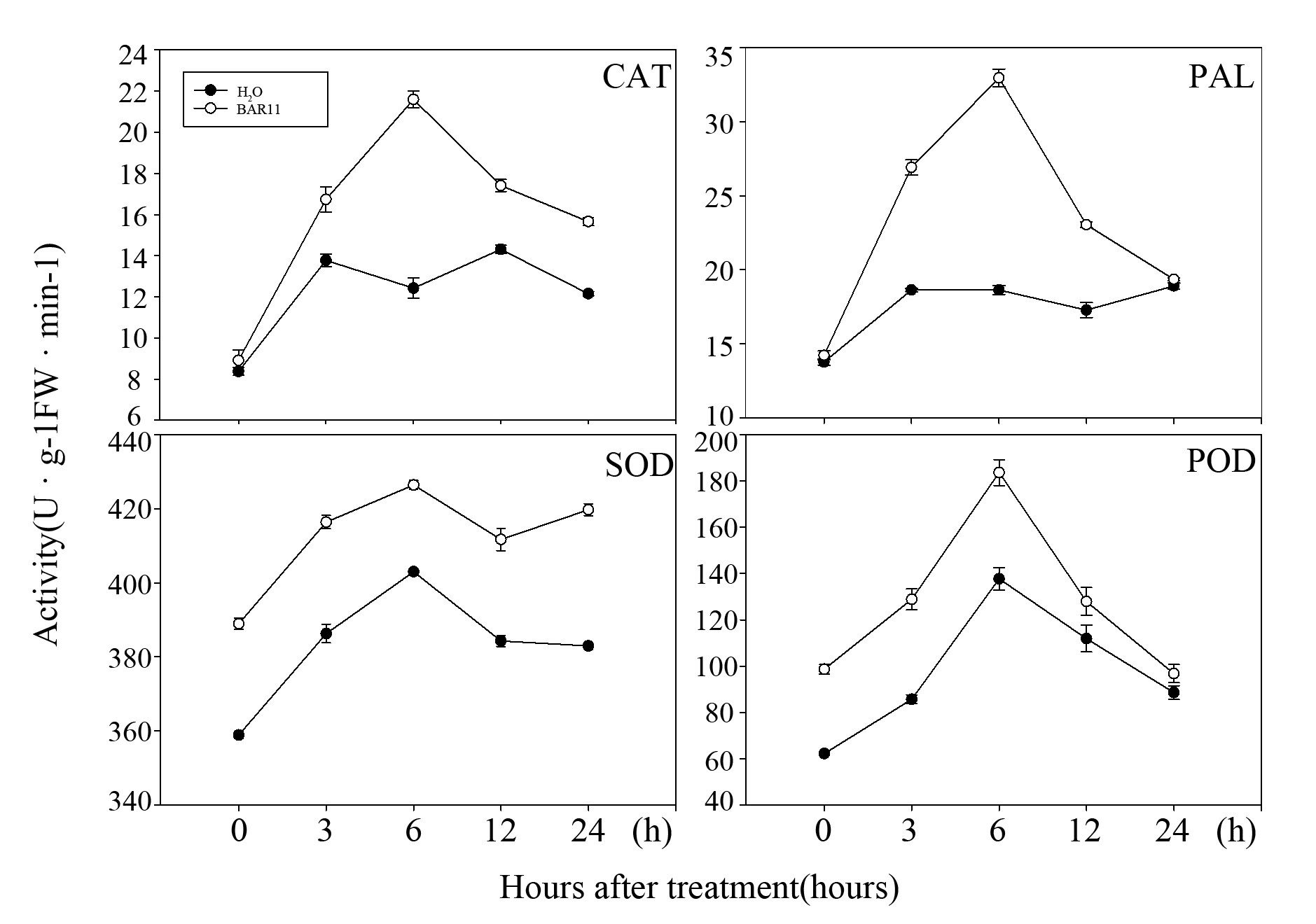

Supplement: Supplementary file 1 [file Image_1.TIF]

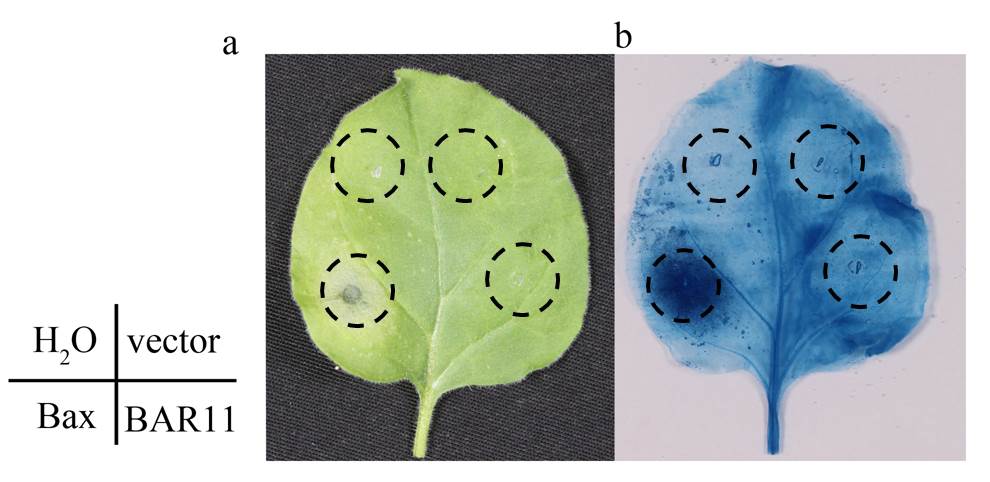

Supplement: Supplementary file 2 [file Image_2.JPEG]
