# Supplementary material for: A Novel Protein Elicitor BAR11 From Saccharothrix yanglingensis Hhs.015 Improves Plant Resistance to Pathogens and Interacts With Catalases as Targets
Source: Front Microbiol. 2018 Apr 9;9:700. doi: 10.3389/fmicb.2018.00700 (PMC5900052; doi:10.3389/fmicb.2018.00700)
Supplement: Supplementary file 3 [file Image_3.PDF]

```
                      *        20         *
BAR11      : -------------------------VLVRRSLVLSSLV :  13
WP_0637412 : ---------------------MSPGGSVRRLFVLLSLV :  17
WP_0788777 : ------------MRGS--------------------TA :   6
SMC97284.1 : ---------------------------MR-VLLIAAV- :   9
ANZ40303.1 : ---------------------------MR-TLLLAAVP :  10
SFQ96232.1 : ---------------------------MRVLLLIAAV- :  10
WP_0531758 : ------------MKRIG-----------LLLALLLAAC :  15
SES44417.1 : ---------------------------MR-ALLIAAL- :   9
SDK06030.1 : ---------------------------MR-ALLIAAI- :   9
SER24664.1 : ---------------------------MR-ALLLTAV- :   9
ACZ86334.1 : ------------MKAA--------TIGAPVLAALTVLL :  18
WP_0788568 : --------------MPDQGDRMTGKAATRVVGALLAA- :  23
AQU66899.1 : ------------MKAV-------RGTAAALLALLVVT- :  18
CCH19455.1 : --------------------------MRAHVAL----L :   8
SCG55021.1 : --------------------------MRTLVSASISLF :  12
WP_0979577 : ------------MRPVNLPPL-HRAAALGLLAALGLGG :  25
SCF09679.1 : --------------------------MRRSAAA--TAL :  10
SNT56505.1 : ------------MKAT--------AVGMPLAALVAALL :  18
GAT67851.1 : ------------MRAS--------AVGAPVLAVVALLL :  18
PIF83899.1 : --------------------------MRALVSASMSLS :  12
SFD08284.1 : --------------------------MRPRLAA---LL :   9
SCF07416.1 : --------------------------MRALVAAPPTLL :  12
WP_0306235 : ------------MNRMNLPKLPHRAAVLGLLGVLALTG :  26
WP_0818981 : ------------MRGQR--------PPMRGLVLAALIF :  18
SEQ93738.1 : ---------------------------MR-VLAIAAV- :   9
WP_0900447 : MARGGHGAAGARLHQPDRQVRTRRPSVVR-ALVVAAV- :  36
WP_0333602 : ---------------------------MAQLAAAVILA :  11
PIG49977.1 : --------------------------MRPRLAA---LL :   9
SMD24479.1 : -----------------------MTVIIRCVLLLLVL- :  14
AGZ44680.1 : ----------------------MIYPQRTAVAG--CLL :  14
SCF04579.1 : ------------------MVGPARRASAWRITGVVVTL :  20
```

```
                40         *         60         *
BAR11      : T---ALVACSPAPPA---------------------TS :  27
WP_0637412 : G---ALSACG-AEPA---------------------PP :  30
WP_0788777 : APAPTSGRT--------------------------AGP :  18
SMC97284.1 : ---LVATGCGAPTGD---------------------TP :  23
ANZ40303.1 : LTAVLLTACGAPVAE---------------------PP :  27
SFQ96232.1 : ---LLATGCGTAAGH---------------------TP :  24
WP_0531758 : PACSSPAGERAPAAHGS---------------HAPSGT :  38
SES44417.1 : ---LVATGCGAPAGD---------------------SP :  23
SDK06030.1 : ---LVATGCGAPAG----------------------SP :  22
SER24664.1 : ---LAAAACGTPAAG---------------------TP :  23
ACZ86334.1 : VARCAAAG---PAAHPA----------AAH---LPPPA :  40
WP_0788568 : ---LLLGGCGGSSDGRA----------------GGDPT :  42
AQU66899.1 : --GCSSAPSVDKKP----------------------AA :  32
CCH19455.1 : TAVVLVAGCGTGSP---------------------GAA :  25
SCG55021.1 : LAALLVTACATGSP--------------------TTTA :  30
WP_0979577 : AAGCSSAADSAPRT-------------------AKVRE :  44
SCF09679.1 : VAVLLVAGCGAGQPP-------------------RDPT :  29
SNT56505.1 : AAGCGGPAS-EHAHHPP----------APS---GQSAV :  42
GAT67851.1 : VARCATADPPHPAAHPAGYPVAAGSGSAAHPAVADPGS :  56
PIF83899.1 : LAALLVTACATGSP--------------------TTTA :  30
SFD08284.1 : AALLLATGCATGTPP-------------------AT-- :  26
SCF07416.1 : IAVLLVAGCAAGSS---------------------DPA :  29
WP_0306235 : AAGCSSAADSTPRTTAD----------------SAPRT :  48
WP_0818981 : AAACTSEAP--------------------------PPP :  30
SEQ93738.1 : ---LVVAGCGATVAA---------------------AP :  23
WP_0900447 : ---LAAAGCGAAAPA---------------------PV :  50
WP_0333602 : TAAAAAAGCAARPADPG-------------------PP :  30
PIG49977.1 : AALLLA-GCATGTPP-------------------AARP :  27
SMD24479.1 : ------AGCGGQVTG---------------------PV :  25
AGZ44680.1 : ALLLGASACDAGTA------------------------ :  28
SCF04579.1 : AATTLAVALTRQTDR--------------------ADQ :  38
```

```
                 80         *       100         *
BAR11      : PATTAPPAGAPTGTAPTAGA------------------ :  47
WP_0637412 : PTTTAPPP------------------------------ :  38
WP_0788777 : AATGVASDGYN--------------------------- :  29
SMC97284.1 : PEG----------------------------------- :  26
ANZ40303.1 : VPQAG--------------------------------- :  32
SFQ96232.1 : PPSAP--------------------------------- :  29
WP_0531758 : HAPSRTHPGAG--------------------------- :  49
SES44417.1 : PASAPPD------------------------------- :  30
SDK06030.1 : PDS----------------------------------- :  25
SER24664.1 : VPT----------------------------------- :  26
ACZ86334.1 : ARETISAPAR---------------------------- :  50
WP_0788568 : PTEDAPRTPDGPSSEDEPADGQESD----AAPEDPEAT :  76
AQU66899.1 : ASTPVQPSPVG--------------------------- :  43
CCH19455.1 : PPTAVPPTAA-----------------------PVAAT :  40
SCG55021.1 : PSTAAPSTAG------------------------LAGS :  44
WP_0979577 : SAPGVSPAPSG--------------------------- :  55
SCF09679.1 : STAATEPPAVD-----------------LAPTRPPTVA :  50
SNT56505.1 : SSPPVVSPASP--------------------------- :  53
GAT67851.1 : AADPVPAPASGPGSGSAPAPGSAPALGSGSGSAPAPEA :  94
PIF83899.1 : PSTAAPSIAG------------------------LAGS :  44
SFD08284.1 : -----GTPP----------------------------- :  30
SCF07416.1 : GTTA--PTAV----------------------APPSVA :  43
WP_0306235 : AEARTSAPPSA--------------------------- :  59
WP_0818981 : PKPVVTVAAYN--------------------------- :  41
SEQ93738.1 : PAGPAPAGSLPAGSLPAGSVQ----------------- :  44
WP_0900447 : APHPVSP------------------------------- :  57
WP_0333602 : PAAPGNPAATG--------------------------- :  41
PIG49977.1 : APLRTGPPPT------------------LASPAGASPA :  47
SMD24479.1 : PEPPASGTG----------------------------- :  34
AGZ44680.1 : --SAPPPAAS---------------------------- :  36
SCF04579.1 : PAAPGSVAAAP--------------------------- :  49
```

```
                          120         *         140         *
BAR11        : --SPTGTGGLSATDTAYAQLAIPQVESALPLLDLVAAR :  83
WP_0637412   : --TTTSAGGLDPTDAAYVQLAIPQAESALPLLDAVRAR :  74
WP_0788777   : -----------ATDLAWAQIMVPMDERTLLLLDLIAGR :  56
SMC97284.1   : ---------LSATDLAFIDLVIPQNESTLAALDLTATR :  55
ANZ40303.1   : ---------PTATDLAFLELVIPQNESALAVLELAGRR :  61
SFQ96232.1   : -------TDLTATDLAFLDLVIPQNESALAVLTLTANR :  60
WP_0531758   : ---------FNATDTAWVQLMIPMIERTLPLLDLAARR :  78
SES44417.1   : ---GLSATDLSATDLAFMDLVIPQNESTLAALDLTASR :  65
SDK06030.1   : ---------LSATDLAFMDLVIPQNESTLAALDLTATR :  54
SER24664.1   : --------DLSATDLAFVDLVIPQNESALAALAL-ADR :  55
ACZ86334.1   : ----TTPGAFNATDVAWLQLMIPMTEQMLRLLELAPEQ :  84
WP_0788568   : GDGDGGIGGLSATDLAWTQLMIPVNERLLPLLETVGER : 114
AQU66899.1   : ------TLSG-ATDAAWIQLMTPMNEGAVELLTLAADR :  74
CCH19455.1   : PSNASSAGAFSPTDIAWLQLTAAMTQRLLPVLDLVPTR :  78
SCG55021.1   : VSPSGSASLFSPTDIAWLQLTVAMNERLLPVLDMVPGR :  82
WP_0979577   : ------TASATPTDIGWVQLMTPMNQQAVKLLTLAAER :  87
SCF09679.1   : PAPRGTPGSFSPTDIAWLQLTVAMAERLLPVLELVPAR :  88
SNT56505.1   : -AASTPSGAFNGTDVGWMQLMIPMDEQLLSMLEMAPKR :  90
GAT67851.1   : VSAGTVPGAFNATDVAWLQLMIPMTEQALDLLVSAPQR : 132
PIF83899.1   : VSPSGSASLFSPTDIAWLQLTVAMNERLLPVLDMVPGR :  82
SFD08284.1   : ------AGQFSATDIAWLQLSVAMAERLLPILDLVPDR :  62
SCF07416.1   : -APVSTAGPFSPTDTAWLQLTVAMAERVLPVLDLVPAR :  80
WP_0306235   : ------TGSASATDVGWVQLMTPMNQQAVKLLTLAAER :  91
WP_0818981   : -----------PTDVAWAQLMAAMNERVLQVLDLAPGR :  68
SEQ93738.1   : --PSPGPAGLSPTDLAFVELVIPQNESALAAVTVAATR :  80
WP_0900447   : ------APELSATDLAFLDLVIPQNETALAALAL--VR :  87
WP_0333602   : ----NTAGDFGGTDIAWVQLMIPMDEQLLPVLDLVAQR :  75
PIG49977.1   : AVLPGTTGQFSATDIAWLQLSVAMAERLLPMLDLVPSR :  85
SMD24479.1   : -----RSTPFGLTERAFVELAIATDEQAVKLLDVGTKQ :  67
AGZ44680.1   : ---SAASSFFGGTDLAWVEINIAMNEELLPLLDLVPAH :  71
SCF04579.1   : ----TAPVVLNGTDDAFIQLLLPMDDGALALIDHLDTR :  83
                         Td a5  6         6  6     r
```

```
                          160         *          180         *
BAR11        : S--GDPALTALVAEVGGGHRAELARLRAVLADAGVAYL : 119
WP_0637412 : A--TDPALAELATRAGVGHRGELTELHGVLERAGATYL : 110
WP_0788777 : A--GDPGLSALARRTSGTHRAELPELRAVLTAAGASGT :  92
SMC97284.1 : P--GS-ALRPVAARLQARYRAELAQVRELLARTGRQES :  90
ANZ40303.1 : E--DS-ALRPVTDQVVTGYRTELARARELLTRAGRQET :  96
SFQ96232.1 : P--GS-ALRPMAAQVEDRYRAELAQVRELLARTGRQES :  95
WP_0531758 : G--TG-GTRGLAAALGRAHRAELDRLRKLRDDAGVSPV : 113
SES44417.1 : P--GS-ALRPVATQLEARYRAELAQVRELLAQNGKQES : 100
SDK06030.1 : P--GS-ALRPVAAWLQARYRAELAQVRELLARTGRQES :  89
SER24664.1 : P--GS-ALGPVVARVGERYRSELAQARELLARAGKAES :  90
ACZ86334.1 : T--SNPRVTRLAARLGAGHRAELPRLRELLGRSGAPGV : 120
WP_0788568 : G--ENPDLRDHATGLVPRVQEEITALRALLEEAGVVYE : 150
AQU66899.1 : V--TDPALRGWASDLAGAHRAELGRMRPLLKELGLPST : 110
CCH19455.1 : T--TDPTWRRLAVEVRASNRADLTRSRQLLGEAGAPTT : 114
SCG55021.1 : T--TDPAWQTFAARLGTAHRADLSTARRLLAESGAPAT : 118
WP_0979577 : A--AEPRVRALAVRLRTGHEAELDRLRPLLTRMGLPRT : 123
SCF09679.1 : T--TDPAWRRLVAQVEATERADLDRARRLLAVAGGPTT : 124
SNT56505.1 : T--SNPEVIQLAKLFAADHRAELLKLRALMDRSGAPKT : 126
GAT67851.1 : A--ADPEVARLAAELDAGHRAQLRTLRGLLKRSGTPPV : 168
PIF83899.1 : T--TDPAWQTFAARLGTAHHADLSIARRLLAESGAPAT : 118
SFD08284.1 : T--TDPAWRRLATRIETSERIHLTRARRLLADFGAPVA :  98
SCF07416.1 : T--TDPAWRRLAAQVEATHRADLTVSRRLLGSSGAPAT : 116
WP_0306235 : A--DEPRLRAFAVRLRAGQEAELDRLRPLLTRMGLPLT : 127
WP_0818981 : T--ADPALADLAGSVGRGHRDELARLRAILTSAKA-GP : 103
SEQ93738.1 : P--GS-ALRPVASRVEAGYRAELAVARELLALAGRQET : 115
WP_0900447 : P--GS-ALRPLADQVAPAYRAELARVREVLTRAGRPES : 122
WP_0333602 : S--ADPALTRLAAQLRPRYASEIEQLRALRARAGLDSA : 111
PIG49977.1 : T--TDPAWRRLATRIETSERTHLTRSRRLLADSGAPVV : 121
SMD24479.1 : A--VQPALKQLANDIGAARRAEVVELHGLLKAAALEYV : 103
AGZ44680.1 : S--TDPEVKALVTEVRAVNDQELATLRALHDEAKLPAE : 107
SCF04579.1 : PSTTDPALRAVLGEIRTTHRAEVVELRGLLAAGNVPEH : 121
                         a        r  6   r 6    g
```

```
                    200         *       220
BAR11      : DEHRGHDMPGMITADEVEAAGRLTGADFDARARALLRA : 157
WP_0637412 : NQHEGHDMPGMVTRDEVVAADRLAGAEFDAEARVLLRR : 148
WP_0788777 : NPHDGMDMKGMVTDEELEAVAASRGAAFDTLARTYLRE : 130
SMC97284.1 : DQHDGHDMPGMITAAELAAVGYAEGTAFDQQLTALLRT : 128
ANZ40303.1 : DLHDGHDMPGMITAAELASIGDSRGSEFDQRLRTLLRT : 134
SFQ96232.1 : DQHDGHDMPGMITAAELTAVGFAEGTAFDQQLKALLRT : 133
WP_0531758 : NVHEGHDMPGMVTASQLDDLRAAGGKAFDELFARRLRE : 151
SES44417.1 : DQHAGHDMPGMITPAEVTAIGYAEGTAFDQQLTALLRT : 138
SDK06030.1 : DQHDGHDMPGMITAAELAAVGYAEGTAFDQQLTALLRT : 127
SER24664.1 : DQHEGHDMPGMITAAELAAVEHVQGADFDLRLKVLLRT : 128
ACZ86334.1 : NVHEGHDMPGMVTAGDLRVLGRTTGAAFDRLFVEHIRE : 158
WP_0788568 : DLHRGHNMPGMVTEEELSALDALDGAAFDEEAVAHLRE : 188
AQU66899.1 : NVHEGHEMPGMVTPGDLTQARAAEGAAFEKVFVIQIRE : 148
CCH19455.1 : NPHEGHDMPGMVTADELAALRSASGKTFHRLLAAHLRA : 152
SCG55021.1 : NPHEGHDMPGMVTEQELTTLRSVTGVAFERLAGQHVRA : 156
WP_0979577 : DVHAGHDMPGMVTEQDLEAAHAAEGPALDRLVLARIRD : 161
SCF09679.1 : NPHEGHDMPGMVTAEQVTALRAATGSPFHRLLAGHLRA : 162
SNT56505.1 : NVHEGHDMPGMVTAADLGVIDQTKGAAFDRLFAKNMRE : 164
GAT67851.1 : NVHEGHDMPGMVTATELAEVNRVEGAAFDRLFTEHVGE : 206
PIF83899.1 : NPHEGHDMPGMVTEQELTTLRSVTGVAFQRLAGQHVRA : 156
SFD08284.1 : NPHEGHDMPGMLTDEELTALRSATGRTFHRLAAGHLRA : 136
SCF07416.1 : NPHEGHDMPGMISADELAALRSATGKAFQRLLAGHLRA : 154
WP_0306235 : DVHAGHDMPGMVTERDLTAARAAEGPAFDRLVLAGIRD : 165
WP_0818981 : NPHEGHDMPGMPTPIVIEAAARAKGKAFDKILVKSLKG : 141
SEQ93738.1 : GLHDGHDMPGMITTAELAALGSARDTAFDQHLEQLLRT : 153
WP_0900447 : DQHDGHDMPGMVTAAELAAAEHAPDP--DDHLKSLLRN : 158
WP_0333602 : NPHAGHRMPGLVDADTLAGIGAAAGAEFDRKAAACLRE : 149
PIG49977.1 : NPHEGHDMPGMVTDEELTALRSATGRAFHHLVAGHLRA : 159
SMD24479.1 : NNHKGHDMPGMPTDEEISALE-ASGPGFDALFAKLLRA : 140
AGZ44680.1 : NPHKGMPMPGMMTPELVAEAAKVRGPAFDKLLLAHLEA : 145
SCF04579.1 : NIHEGHQMPGMVTDERLADLRATPDAEVPSRAVGLLRA : 159
               H Gh MpG6     6       g  f       6
```

```
              *       240         *       260
BAR11      : HFEESATVARAELAAGSDAALLALAGDLDGARRGYLAK : 195
WP_0637412 : HFEESAAVARSELAAGADAGLLELTGRIERARADYLSK : 186
WP_0788777 : HFEQSLVVARGETHSGASASAKRLAAELTKRRTGQLAA : 168
SMC97284.1 : QFEEARTVARAELSSGTSTPVVELSARIDSTRAEFLAL : 166
ANZ40303.1 : QFEEARTVARAELSGGTSPPVLELGTRIERTRAEFLAL : 172
SFQ96232.1 : QFEEARTVARAELSSGTSKPVLELGARVDSTRAEFLAL : 171
WP_0531758 : HLDQSLKVTRGERGSGSDPRALALAADLVRSRTGQLAQ : 189
SES44417.1 : QCEEARTVARAELSSGTSKPVVELSARIVAARAEFLTL : 176
SDK06030.1 : QFEEARTVARAELASGTSTPVVELSARIDSTRAEFLAL : 165
SER24664.1 : QFEEARTVARAELASGTSKPVLELGERIDSSRAEFLEL : 166
ACZ86334.1 : HLEQGILVSRGEQGSGAEQAVRELAADIERTRAAQLAL : 196
WP_0788568 : FLEETASVSRSETDAGSHPGTIALAADLDRVRTEQIAE : 226
AQU66899.1 : HLEHSARVSRSEVDAGSEAAARKLAASLVEARRGELAG : 186
CCH19455.1 : YLTQTTRVATAEQKSGANPATTALATTVITTNQTHLNN : 190
SCG55021.1 : HLQQAVRIAAAEQRNGAYPATTALAAEVARAGDAELTR : 194
WP_0979577 : HLRQSAQVSRSEITAGGRADARELARALVTAREGFLAE : 199
SCF09679.1 : HLTQAARIAGAEQHAGAHPATTAMAGAVVRDGNAHLAR : 200
SNT56505.1 : HLEQGILLCRGEQGAGADQATRELAGNIEKARAAQLDR : 202
GAT67851.1 : YLRQSVQVARGEQGSGADRETRAFAAAVERTRAGELIR : 244
PIF83899.1 : HLQQVVRVATAEQRNGVHPATTALAAEVVRAGDAELTR : 194
SFD08284.1 : HLTQSVRIASAEQQGGSDPAATGLAATVVRAGTADLAR : 174
SCF07416.1 : HLTQAVRIAAAEQRGGVHPETIALAAAVVRAGTTDLAR : 192
WP_0306235 : HLRQSAQVSRSEITAGGRADARELARALVAARETFLAE : 203
WP_0818981 : HLDQSAMVTASEQKAGAAREALDLAADMTAARTEQLAA : 179
SEQ93738.1 : QFEEARTVARAELSSGKSAPALELGARIERTRAEFLAL : 191
WP_0900447 : QFEEARTVARSALSAGTNATVVELGGCIDKTRETYLAT : 196
WP_0333602 : HLDQLASLARSELSNGTSASVKDLARQVVESRSAGLAA : 187
PIG49977.1 : HLTQSVRIATAEQRGGFDPATTALAAAVVRAGAADLAR : 197
SMD24479.1 : HLDESITVVRSAAQAVSDEPTRALARRMETDRIGFTQR : 178
AGZ44680.1 : HFDQGVRLAGSEEKSGLEPRTKALAGEVIGTRKKYLPK : 183
SCF04579.1 : HLEQSVVLSRGEQTAGGSPEVRALAGRIEATRAAELSA : 197
                    6   e   g           6
```

```
                 *        280          *        300
BAR11      : LSGS---------------------------------- : 199
WP_0637412 : LSVS---------------------------------- : 190
WP_0788777 : LRALDG-------------------------------- : 174
SMC97284.1 : LEDAS--------------------------------- : 171
ANZ40303.1 : L------------------------------------- : 173
SFQ96232.1 : LGDAPRSP------------------------------ : 179
WP_0531758 : LARLGALASATS-------------------------- : 201
SES44417.1 : LKDAS--------------------------------- : 181
SDK06030.1 : LEDAS--------------------------------- : 170
SER24664.1 : LGGSRQ-------------------------------- : 172
ACZ86334.1 : LDG----------------------------------- : 199
WP_0788568 : LERVIG-------------------------------- : 232
AQU66899.1 : LERLTA-------------------------------- : 192
CCH19455.1 : LP------------------------------------ : 192
SCG55021.1 : LDRLTL-------------------------------- : 200
WP_0979577 : LERLPGAVRALG-------------------------- : 211
SCF09679.1 : LDRLDPG------------------------------- : 207
SNT56505.1 : LDG----------------------------------- : 205
GAT67851.1 : LTGLGGASG----------------------------- : 253
PIF83899.1 : LDRLTP-------------------------------- : 200
SFD08284.1 : LDHLDRAPTLPPVPAARQ-------------------- : 192
SCF07416.1 : LDRLDPGVDEIRGSMPVICLTRPSTATGEHTAGDAQAA : 230
WP_0306235 : LARLPGAERALG-------------------------- : 215
WP_0818981 : LSGIAR-------------------------------- : 185
SEQ93738.1 : LDAS---------------------------------- : 195
WP_0900447 : LNAL---------------------------------- : 200
WP_0333602 : LPAGASGAAGGG-------------------------- : 199
PIG49977.1 : LDHLDRAIDASPGPA----------------------- : 212
SMD24479.1 : LGAVVPV------------------------------- : 185
AGZ44680.1 : VAG----------------------------------- : 186
SCF04579.1 : LAGRPGATGDADN------------------------- : 210
             6
```
